# Necessity of long-term support after disasters: lessons from disaster-related death criteria after the Fukushima nuclear disaster

**DOI:** 10.7189/jogh.15.03024

**Published:** 2025-06-13

**Authors:** Chika Yamamoto, Toyoaki Sawano, Saori Nonaka, Yuna Uchi, Moe Kawashima, Masaharu Tsubokura

**Affiliations:** 1 Department of Radiation Health Management, Fukushima Medical University School of Medicine, Fukushima, Japan; 2 Department of Surgery, Jyoban Hospital of Tokiwa Foundation, Iwaki, Fukushima, Japan; 3 Jyoban Hospital of Tokiwa Foundation, Iwaki, Fukushima, Japan; 4 Shirakawa Kosei General Hospital, Shirakawa, Fukushima, Japan

## Abstract

As disasters increase in both frequency and complexity, their indirect effects – such as disruptions to medical care, environmental changes, prolonged periods of evacuation, and psychosocial stress – are increasingly recognised as critical public health issues. In Japan, disaster-related deaths (DRDs) represent an institutional framework to address these secondary health impacts. We compare two DRD certification systems: the Nagaoka Criteria and the Minamisoma Criteria. The former employs a strict time-based cutoff, often excluding deaths beyond six months post-disaster, whereas the latter emerged from the Fukushima disaster context and allows for broader, individualised assessments, recognising long-term and indirect health deterioration as disaster-related. These differences highlight the importance of flexible criteria that reflect the realities of prolonged displacement and chronic disease progression. By leveraging DRD data, it is possible to identify populations most vulnerable to secondary health impacts and incorporate these insights into disaster preparedness and health policy. As promoted by the Sendai Framework’s ‘Build Back Better’ principle, revising DRD certification to encompass long-term impacts is essential to reduce preventable disaster-related mortality and strengthen health systems in the aftermath of complex emergencies.

In recent years, the increasing frequency of disasters has necessitated strengthening disaster risk reduction efforts based on past disaster experiences at the regional level [1]. Disasters exert diverse impacts on individuals and communities. One significant impact is indirect effects, including changes in living conditions post-evacuation, environmental changes, reduced access to medical care, and psychosocial stress [2–4]. These effects are particularly pronounced among vulnerable populations during disasters, such as the older adults, individuals with disabilities, and pregnant women, highlighting the critical importance of public health interventions targeted towards these groups.

In worst cases, the indirect health effects of disasters can result in excess mortality. In contrast to direct deaths [5], which are caused immediately by the disaster itself, these cases are classified as indirect deaths [6]. Numerous such deaths have been reported following disasters such as hurricanes in the USA [2] and the Great East Japan Earthquake in Japan [6,7]. The methods for identifying indirect deaths vary across countries. In the USA, the Centers for Disease Control and Prevention classify indirect deaths [8] based on death certificates and autopsy reports. In contrast, although Europe experiences frequent flood-related disasters, no standardised approach has been established for classifying disaster-related deaths (DRDs) [9]. In Japan, the concept of DRDs was introduced following the 1995 Great Hanshin-Awaji Earthquake [7,10], when a legal framework was established to ensure condolence payments to bereaved families. In general, DRDs are determined based on applications submitted by bereaved families, where a special Disaster Condolence Payment Review Committee recognises deaths caused by physical and psychological burdens during evacuation or the exacerbation of pre-existing conditions [6].

The standard certification criteria for DRDs in Japan were first established as the Nagaoka Criteria [11] following the 2004 Niigata Prefecture Chuetsu Earthquake. However, in Minamisoma City, Fukushima Prefecture, where residents were forced to evacuate following the 2011 Great East Japan Earthquake and the Fukushima Daiichi Nuclear Power Plant accident, the existing criteria were found to be insufficient, leading to the development of the Minamisoma Criteria [12]. Here we compare the two criteria which had emerged from two distinct disaster contexts to derive insights into disaster response and public health challenges.

Two significant differences between the Nagaoka Criteria and the Minamisoma Criteria are evident (**Figure 1**). The first pertains to the timeframe between the disaster and death. The Nagaoka Criteria establish a temporal standard, presuming that ‘if more than six months have passed since the disaster, the death is not considered disaster-related’. In contrast, the Minamisoma Criteria do not impose a time limitation, adopting a more flexible perspective that ‘if the disaster is considered to have even slightly hastened the time of death, it may be recognised as disaster-related’. The second difference concerns the consideration of individual circumstances. The Minamisoma Criteria emphasise an individualised assessment based on application forms submitted by bereaved families and from medical records. Even for common diseases such as pneumonia, myocardial infarction, and heart failure, the determination is based on whether the disease progression would have followed the same course in the absence of the disaster. In the Minamisoma Criteria, DRDs are recognised based on whether evacuation and subsequent living conditions exacerbated chronic health risks and psychological burdens. In contrast, the Nagaoka Criteria focus on whether the individual was already at high risk before the earthquake, stating that cases involving ‘patients who were not at high risk before the earthquake’ or ‘those with symptom improvement leading to repeated hospitalisations’ would not be recognised as DRDs. There is no explicit consideration of individual circumstances following the earthquake within the Nagaoka Criteria.

**Figure 1:**
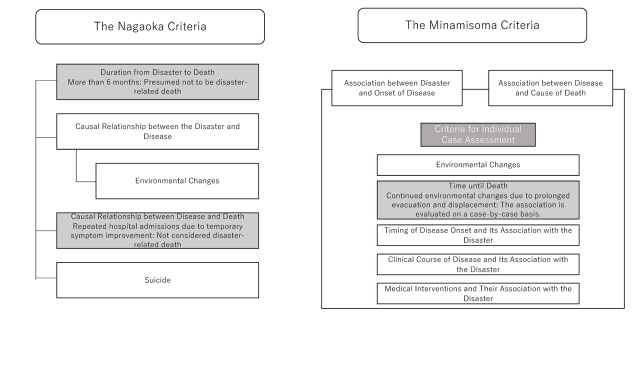
Conceptual differences between the Nagaoka Criteria and the Minamisoma Criteria.

The Minamisoma Criteria, which do not impose a temporal restriction on DRD certification, play a crucial role in understanding long-term health impacts following disasters and establishing appropriate support systems. In Ishinomaki City, Miyagi Prefecture, the median duration from the disaster to death was 24 days, with approximately 80% of DRDs occurring within three months of the disaster [6,13]. In contrast, in Minamisoma City, where prolonged evacuation was necessary due to Great East Japan Earthquake and the Fukushima Daiichi Nuclear Power Plant accidents, the average time from the disaster to death was 230.6 days, with 37.8% of cases being certified more than 6 months post-disaster [14]. Repeated evacuations [15] and changes in living conditions exacerbate both physical and psychological burdens on disaster victims, contributing to deteriorating health conditions [16]. Additionally, restricted access to medical care has been reported as a significant factor, with evacuees experiencing the loss of primary care physicians, missed opportunities sfor routine screenings such as cancer check-ups [17,18], and an increased risk of delayed treatment [19]. These factors complicate the management of chronic diseases and accelerate the progression of pre-existing conditions, leading to mortality. Furthermore, environmental changes due to evacuation may lead to the collapse of local communities [20], contributing to cognitive decline and overall deterioration in health status. As ageing populations continue to grow, evaluating and addressing the long-term health impacts of disasters has become an urgent public health challenge.

Moreover, the Sendai Framework for Disaster Risk Reduction [21] (2015–30), an international disaster resilience initiative, advocates for the ‘Build Back Better’ principle, emphasising the importance of long-term health support post-disaster. Within this initiative, comprehensive risk assessments that account for potential cascading disasters are required to deepen the ‘understanding of disaster risk’. Large-scale disasters can lead to long-term health consequences as evidenced by cases such as the Fukushima Daiichi Nuclear Power Plant accident and Hurricane Katrina. However, research on indirect deaths caused by these disasters and efforts to categorise their health impacts systematically remain insufficient.

In Japan, leveraging DRDs records to evaluate indirect health effects and integrate findings into future disaster mitigation measures is essential. This necessitates the development of DRDs certification standards that account for long-term health impacts as well as the establishment of a framework capable of accurately assessing ‘indirect deaths’. Given this context, it is necessary to use DRDs data to clarify the characteristics of disaster victims who are particularly vulnerable to secondary health impacts. Furthermore, incorporating these data into disaster preparedness planning, as well as medical and welfare policies, could minimise disaster-related health consequences and facilitate the establishment of more effective support systems.
